# The Role of the Farmer as an Eugenist

**Published:** 1915-08

**Authors:** 

**Affiliations:** San Antonio


### The Role of the Farmer as an Eugenist.

Because of the customary conception of the farmer as a tiller of the soil it may sound a little grotesque to class him as an eugenist. But such he is. For is not he the salt of the earth? More than that, is not the farm the cradle of industry? What would become of the teeming millions living today but for the products of the farm? Let the farmer fail to produce and the world would cease its struggle, just as the clock that has lost its pendulum. More than that, the farm is the check for over-civilization, the refuge of misspent ambition, the hearthstone of our living principles, and the source of our national vitality. When vanity and ambition has spent our physical strength, when the luxuries of life and the essentials have been lost to us then we turn back to Mother Earth in search of the necessities and feel proud to drink from her abundance. Indeed, it has been said: "If any durable good is to come to us as a people from the carnage of the Old World, one of the gains will be quickened appreciation of our dependencies on the essentials of the earth and an accelerated determination to return to them as to the mother that gave us birth, and to the things that were ordained to us in the beginning. Every experience that brings us back to the munificence of the earth and to a conscious dependence on it, brings us back necessarily to the farmer, and he is elevated in the essential plan of any enduring human society. When the armies have killed each other off, when the supplies shall have been exhausted, when the military organizations will have tired of their vanities, when vengeance has been spent, and when society becomes ashamed of itself, then we shall begin all over again at a slow and laborious process of reconstruction: and we must begin on the earth."

The farmer has learned the struggle for existence better than the theorist himself. He knows that the principle of life is to live and let live; that victory is not to the strong; that the smallest and most fragile living things exist beside the most ferocious; he has learned, in fact, that the whole contrivance of nature is to protect the weak. The world has never been conquered by force; the true heroes are those who silently conquer the laws of nature. In so doing, only, can man conquer the world. The struggle for existence means more than anything else the dependence of one individual on another; not only the life of the individual, but the success of leaving progeny. It is not the survival of the fittest but the ethically best.

The farmer, again, knows the significance of practical eugenics. He has learned that no real value can be obtained unless by select breeding. He has learned that the highest quality in his stock or grain is the result of rational selection and control of the laws that make development possible. Thus it is that the farmer becomes the bone and sinew of society—our ideal eugenist.

Because of his practical knowledge of these facts he is the better prepared to understand the more complex problem of improving human kind; and, it will only be necessary to present this matter clearly to him to have his full co-operation.

